# Successful Use of Bortezomib in an Adolescent with Refractory TTP

**DOI:** 10.1155/2023/8173903

**Published:** 2023-11-25

**Authors:** Junaid Ahmad Wali, Brian M. Quigley, Beverly Schaefer

**Affiliations:** ^1^Department of Internal Medicine, University at Buffalo, Jacobs School of Medicine and Biomedical Sciences, Buffalo, New York, USA; ^2^Division of Behavioral Medicine, Department of Medicine, University at Buffalo, Jacobs School of Medicine and Biomedical Sciences, Buffalo, New York, USA; ^3^Department of Pediatrics, University at Buffalo, Jacobs School of Medicine and Biomedical Sciences, Buffalo, New York, USA

## Abstract

With increasing early and upfront use of rituximab and caplacizumab in the modern management of immune-mediated thrombotic thrombocytopenic purpura (iTTP), the risk of refractory disease is expected to decline. However, despite the use of adequate initial therapy, a small subset of patients develop a refractory disease which is difficult to manage. Bortezomib has come to be known as a safe and effective treatment option for refractory iTTP, but its use in children is limited. Here, we describe the case of an adolescent patient with refractory iTTP who had a satisfactory and sustained response to the use of bortezomib.

## 1. Case Presentation

An 18-year-old female patient presented to a tertiary pediatric hospital with a two-week history of diffuse petechial rash and an acute episode of self-resolving left-sided facial droop and weakness. The patient had been medically healthy and there was no recent history of diarrhea, upper respiratory tract infection, fever, other systemic complaints, or new medications.

Initial laboratory work was significant for a low platelet count of 11 × 10^9^/L with low hemoglobin of 6.6 gm/dl and 2+ schistocytes on the peripheral smear. Serum creatinine was 0.8 mg/dL. Initial screening evaluation for autoimmune disease, HIV, pregnancy, and malignancy was negative.

Due to the high PLASMIC score of 7 and the presence of schistocytes, a presumptive diagnosis of immune-mediated thrombotic thrombocytopenic purpura (iTTP) was done. The patient was started on 1× volume plasma exchange (PEX) and high-dose steroids (10 mg/kg/day × 3 days, followed by 2.5 mg/kg/day on the subsequent days), with the addition of rituximab at a dose of 375 mg/m^2^ weekly × 4 on day 3. On day 5, ADAMTS13 level from admission resulted as undetectable, with an antibody titer of 64 units/ml (normal <12) confirming the diagnosis of iTTP. Despite five days of treatment with 1× volume PEX, her platelet count continued to be below 50 × 10^9^/L, which was consistent with that of a refractory disease. The volume of plasma exchange was increased to 1.5× starting on day 6 and caplacizumab was added to the regimen starting day 10.

On day 11, the patient achieved a clinical response with normalization of platelet count and LDH, and the plasma exchange was stopped [1]. The patient was discharged home on hospital day 16 to continue a steroid taper and daily caplacizumab with the plan to receive subsequent doses of rituximab as an outpatient.

**Figure 1:**
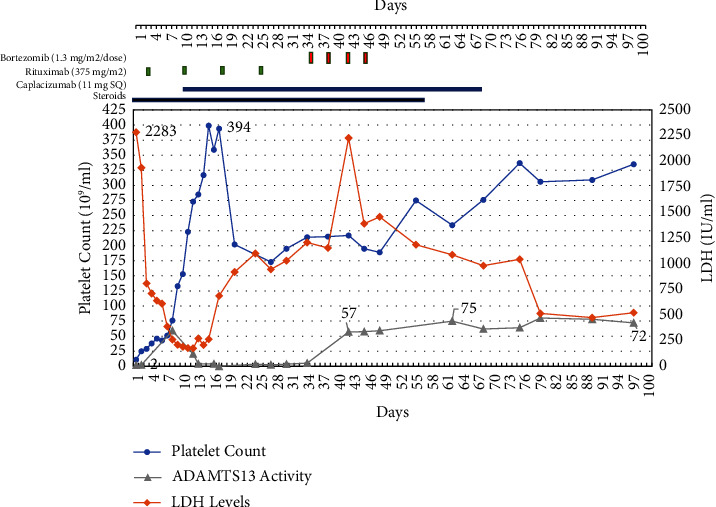
Change in platelet count and serum LDH and ADAMTS13 levels over time with different treatment modalities.

During her outpatient follow-up, the patient continued to be in clinical remission but her ADAMTS13 levels remained persistently low despite evidence of B-cell aplasia on lymphocyte flow cytometry. The patient was prescribed caplacizumab therapy for an additional 28 days. Due to the concern for a high risk for relapse in the setting of persistently low ADAMTS13, the patient was started on bortezomib at a dose of 1.3 mg/m^2^/dose on day 36 followed by 3 additional doses on days 38, 42, and 45. On day 42, one week after initiating plasma cell-directed therapy with bortezomib, the patient achieved complete ADAMTS13 remission with ADAMTS13 levels rising to 57% (Figure 1).

The patient had a transient mild local skin reaction to bortezomib but did not have any symptoms of peripheral neuropathy. At two years follow-up, the patient continues to be in remission with normal platelet count, LDH, and ADAMTS13 levels and no signs of underlying autoimmune disorder or immune dysregulation.

## 2. Discussion

Refractory idiopathic thrombotic thrombocytopenic purpura is defined as the lack of platelet response after 4 to 7 days of initial treatment with plasma exchange (PLEX) and steroids [1]. With the increasing upfront use of rituximab and caplacizumab, the incidence of refractory disease will continue to decline. However, the persistent production of the ADAMTS13 antibodies by the plasma cells could be a harbinger of the disease relapse in individuals who initially respond but continue to have persistently low ADAMTS13 levels on outpatient monitoring [2].

With a better understanding of the pathophysiology of the disease and advances in treatment, the therapeutic landscape of iTTP management continues to evolve. Therapeutic plasma exchange (PEX) and steroids continue to be the cornerstone of therapy with increasing data accumulating regarding the efficacy of the upfront use of rituximab and caplacizumab to help reduce the duration of PLEX as well as to decrease the incidence of refractory disease and relapse [3–7].

Possible risk factors for refractory disease in patients with iTTP include neurological involvement at the time of presentation, elevated cardiac enzyme levels, and persistently low ADAMTS13 levels 3 to 7 days after the initiation of plasma exchange [6].

Various treatment approaches have been utilized with varying levels of success in individuals with refractory diseases or those with persistently low ADAMTS13 levels, but data are typically limited to small case series and case reports [7–10]. In these patients, clinicians must balance treatment considerations including time to efficacy, patient comorbidities, medication side effects, health insurance authorizations, and potential costs.

Over the last few years, there have been quite a few case reports and case series of the successful use of bortezomib in patients with refractory iTTP and it has come to be regarded as a safe and effective treatment option in this patient population [11–15]. Bortezomib, a proteasome inhibitor, is FDA-approved for use in multiple myeloma as well as mantle cell lymphoma. Bortezomib functions through a selective inhibition of proteasomes, leading to cell cycle arrest and apoptosis of plasma cells. In patients with iTTP who continue to have refractory diseases following rituximab administration, it may be beneficial to use bortezomib to eliminate plasma cells and the remaining site of production of anti-ADAMTS13 antibodies. Bortezomib also induces apoptosis and inhibits maturation in dendritic cells, which has been shown to be a possible alternative mechanism of action for its beneficial effects in patients with relapsed/refractory TTP.

Possible side effects associated with the use of bortezomib include injection site reactions, neurologic side effects including peripheral neuropathy, motor weakness, cardiac toxicity, and abdominal complaints including abdominal pain, anorexia, and constipation. The most feared complication of bortezomib can be neuropathy, which can be long-standing and severe. Despite a growing body of evidence to support the use of bortezomib in refractory or relapsed iTTP, the concern for possible side effects, particularly peripheral neuropathy, has typically restricted its use to the small fraction of patients with refractory disease [16].

Our case report highlights the complexity involved in the management of the disease that was initially refractory but did respond to the addition of rituximab and caplacizumab. At the same time, due to the persistently low levels of ADAMTS13, the patient was at risk for disease relapse. The use of bortezomib, a proteasome inhibitor with activity against antibody-producing plasma cells, helped improve the levels of ADAMTS13 back to normal within a week of the start of the therapy in this patient.

Another plasma cell-directed therapy, daratumumab, has also been found to be efficacious in patients with refractory iTTP [9, 17]. Daratumumab binds to CD38 receptors on plasma cells and induces cell apoptosis via antibody-dependent cellular toxicity, complement-dependent cytotoxicity, and antibody-dependent cellular phagocytosis. Daratumumab has been shown in case reports to reduce the incidence of iTTP relapse in patients with persistent ADAMTS13 deficiency and has been used in refractory or frequently relapsing TTP [9, 17].

Given the rarity of iTTP in pediatrics, pediatric hematologists/oncologists generally have limited experience in treating iTTP, and even less familiarity with treatment for patients with persistent ADAMTS13 deficiency or relapsed or refractory disease.

Our case report highlights the safety as well as efficacy of bortezomib in a pediatric patient with refractory TTP. It also highlights the use of serial ADAMTS13 monitoring as an important tool to predict the risk for disease relapse and rapid and sustained improvement in ADAMTS13 levels following just two doses of bortezomib. This therapy was well tolerated in our adolescent patient who continues to have normal ADAMTS13 levels for more than 24 months from diagnosis at the time of writing this report.

For iTTP patients with persistently low ADAMTS13 levels following upfront therapy, plasma cell-directed therapy including bortezomib and daratumumab represents a promising treatment option to help reduce the risk of disease relapse.
